# Neutrophil Gelatinase-Associated Lipocalin (NGAL) Predicts Response to Neoadjuvant Chemotherapy and Clinical Outcome in Primary Human Breast Cancer

**DOI:** 10.1371/journal.pone.0045826

**Published:** 2012-10-09

**Authors:** Antonia Sophie Wenners, Keyur Mehta, Sibylle Loibl, Hyerim Park, Berit Mueller, Norbert Arnold, Sigrid Hamann, Joerg Weimer, Beyhan Ataseven, Silvia Darb-Esfahani, Christian Schem, Christoph Mundhenke, Fariba Khandan, Christoph Thomssen, Walter Jonat, Hans-Juergen Holzhausen, Gunther von Minckwitz, Carsten Denkert, Maret Bauer

**Affiliations:** 1 Department of Gynecology and Obstetrics, University Medical Center Schleswig-Holstein, Kiel, Germany; 2 German Breast Group, GBG ForschungsGmbH, Neu-Isenburg, Germany; 3 Department of Obstetrics and Gynaecology, Rot-Kreuz-Klinikum Muenchen, Munich, Germany; 4 St. Markus Hospital, Frankfurt am Main, Germany; 5 Translational Tumorpathology Unit, Institute of Pathology, Charité, Berlin, Germany; 6 Department of Gynecology, Martin-Luther-University of Halle-Wittenberg, Halle, Germany; 7 Department of Pathology, Martin Luther University of Halle–Wittenberg, Halle, Germany; University of Texas MD Anderson Cancer Center, United States of America

## Abstract

In our previous work we showed that NGAL, a protein involved in the regulation of proliferation and differentiation, is overexpressed in human breast cancer (BC) and predicts poor prognosis. In neoadjuvant chemotherapy (NACT) pathological complete response (pCR) is a predictor for outcome. The aim of this study was to evaluate NGAL as a predictor of response to NACT and to validate NGAL as a prognostic factor for clinical outcome in patients with primary BC. Immunohistochemistry was performed on tissue microarrays from 652 core biopsies from BC patients, who underwent NACT in the GeparTrio trial. NGAL expression and intensity was evaluated separately. NGAL was detected in 42.2% of the breast carcinomas in the cytoplasm. NGAL expression correlated with negative hormone receptor (HR) status, but not with other baseline parameters. NGAL expression did not correlate with pCR in the full population, however, NGAL expression and staining intensity were significantly associated with higher pCR rates in patients with positive HR status. In addition, strong NGAL expression correlated with higher pCR rates in node negative patients, patients with histological grade 1 or 2 tumors and a tumor size <40 mm. In univariate survival analysis, positive NGAL expression and strong staining intensity correlated with decreased disease-free survival (DFS) in the entire cohort and different subgroups, including HR positive patients. Similar correlations were found for intense staining and decreased overall survival (OS). In multivariate analysis, NGAL expression remained an independent prognostic factor for DFS. The results show that in low-risk subgroups, NGAL was found to be a predictive marker for pCR after NACT. Furthermore, NGAL could be validated as an independent prognostic factor for decreased DFS in primary human BC.

## Introduction

Human neutrophil gelatinase-associated lipocalin (NGAL or lipocalin 2) is a small 25 kDa extracellular protein, expressed by neutrophils and originally presenting itself in complex with neutrophil gelatinase, also known as matrix metalloproteinase 9 (MMP-9) [1]. NGAL belongs to the lipocalin protein family, which has been classified as transport proteins of lipophilic molecules. As an acute phase protein, NGAL additionally plays a role in inflammatory conditions and immune response, including the synthesis of prostaglandins [2]. It has also been observed that NGAL actively participates in the process of proliferation, developement and differentiation of different human tissues [3], [4]. Thus, NGAL plays an important role in the pathophysiology of neoplasias. Regarding different tumor entities, contradicting results about its involvement in tumor developement were shown. Whereas NGAL seems to have a pro-tumoral effect in breast [5], [6], stomach [7], [8], oesophagus [9], kidney [10] and thyroid cancer [11], its influence on ovary [12] and pancreas [13] appears to be rather anti-tumoral. For colorectal cancer results are controversial [14], [15], [16]. These findings suggest a neoplasia-specific effect of NGAL. Stoesz et al. [17] observed that NGAL was overexpressed in breast cancer. Based on these findings, we showed in our previous work that in breast cancer NGAL expression is correlated with negative hormone receptor (HR) status, human epidermal growth factor receptor 2 (HER2) overexpression, poor grading and positive nodal status. NGAL expression was associated with shorter disease-specific and disease-free survival and was proven to be an independent prognostic marker for disease-free survival [5].

Neoadjuvant chemotherapy (NACT) is used for treatment of locally advanced breast cancer since the 1970s in order to downsize large tumors to enable breast-conserving surgery [18]. Lately, NACT is increasingly being used for treatment of early-stage breast cancer as well [19]. NACT reaches at least equivalent disease-free survival (DFS) and overall survival (OS) rates compared to adjuvant chemotherapy, presumably through early treatment of systemic micrometastatic disease [18], [20]. An advantage of NACT is that it gives information about tumor response to a specific chemotherapeutic regimen and therefore allows biologic studies to investigate molecular determinants of chemotherapy response. It was shown that tumor response to preoperative chemotherapy correlates with outcome. Pathological complete response (pCR) seems to be the most powerful predictor of response and survival [21]. 3–30% of patients achieve pCR after NACT and have improved outcome (DFS and OS) compared to patients with residual disease at the primary tumor site or lymph nodes [22], [23]. Despite high response rates of 60–90% to NACT [20], a small population fails to respond or show progressive disease and therefore features poor prognosis. [24]. Early identification of these non-responders is an urgent goal to enable alternative treatment choices. There are already several predictive biological markers such as negative steroid receptor status, high histopathological grading, high Ki67-proliferation index [25], small tumor size [26] and tumor type of invasive ductal carcinoma (IDC) [27]. Lately, four subgroups of breast cancer have been identified based on gene expression profiles (luminal A and B, basal-like and HER2 positive) [28]. Even if detection of predictive markers strongly depends on the drugs used in NACT [29], [30], one of the largest studies on gene expression showed that specific gene expression profiles are valid independent variables predicting pathological complete response [31]. Therefore, it should be ultimated ambition to find more reliable markers that can predict clinical or pathological response in early stage of treatment. NACT allows clinical monitoring of in vivo tumor responses and therefore presents an interesting model to evaluate new biological markers [25]. Information about such markers could help to perform an individual and optimal treatment concept for each patient, a so called “tailored therapy”.

NGAL could be one of those potential biomarkers to forecast response to NACT. Hence, based on our previous work we aim to not only validate NGAL as a predictor of prognosis in breast cancer, but also to evaluate NGAL as a potential predictive marker in neoadjuvant chemotherapy.

## Materials and Methods

Specimens and clinical information were provided by the neoadjuvant GeparTrio study, a prospective, multicentre, randomized phase III trial, that investigated a total of 2090 patients with operable primary breast carcinoma (cT2-4, cN0-3, M0) between July 2001 and December 2005 [32], [33], [34]. Ethics approval for the study was obtained from the ethics committee of each participating institution. All patients gave their written informed consent for participation in the study and for tumor tissue sampling. The trial registration number (clinicaltrials.gov) is NCT 00544765 [32], [33]. Primary endpoint of the GeparTrio trial was to evaluate pCR after neoadjuvant cytotoxic therapy with six to eight cycles docetaxel, doxorubicin, cyclophosphamide (TAC) or two cycles of TAC followed by four cycles of vinorelbine and capecitabine, depending on response status [33]. pCR for this analysis was defined as no residual invasive tumor cells from the breast and axillary tissue (ypT0/ypTis, ypN0). A total of 855 breast cancer cases with corresponding clinical and histopathological data, such as analyses of estrogen receptor (ER), progesterone receptor (PR) and HER2 status, histological grading and subtype, lymph node status and tumorsize, were available for this study. All data, including clinical and pathological response, as well as follow-up data were provided by the German Breast Group (GBG). The median follow up time was 59 months with a range between 2 months and 96 months. The median age was 51 years, ranging from 24 to 78 years.

### Pathologic assessment

**Figure 1:**
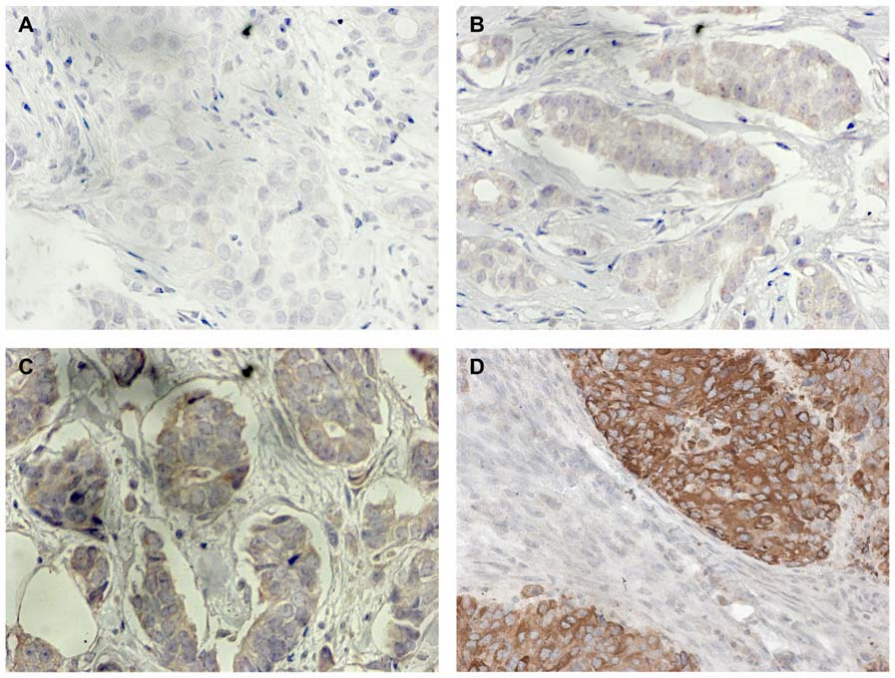
NGAL expression in human breast cancer tissues. (A) Negative NGAL staining (staining intensity score 0). (B) Weak NGAL staining (staining intensity score 1). (C) Moderate NGAL staining (staining intensity 2). (D) Strong NGAL staining (staining intensity 3). Original magnification: 400×.

Tissue microarrays of formalin-fixed, paraffin-embedded pretherapeutic core biopsies were constructed by the Institute of Pathology, Charité University Hospital, Berlin, Germany. Immunohistochemical staining was performed by Discovery XT staining system (Ventana Medical Systems, Tuscon, USA). After epitope retrieval, primary anti-NGAL antibody was added in a dilution of 1∶120. The generation of this rabbit polyclonal anti-NGAL antibody has been reported by Stoesz et al. [17]. After incubation with the secondary polyclonal goat anti-rabbit antibody in a dilution of 1∶200 (DAKO, Denmark A/S) antibody labelling was visualized using the ABC vector stain kit (Vector laboratories, Burlingame, CA). NGAL was evaluated by expression (negative vs positive) and intensity of staining. The intensity score ranged from 0 (no staining), 1 ( = weak), 2 ( = intermediate) to 3 ( = strong) staining (Figure 1) [35]. Scoring of all slides was done by two independent investigators (H.P. and M.B.).

### Statistical analyses

The primary clinical endpoints were pCR, disease-free survival and overall survival. Survival was calculated in months from the date of diagnosis until the date of first relapse (DFS) or death (OS) for each patient. Both DFS and OS time were censored at the date of last follow-up if no recurrence, respectively death was observed.

Patient characteristics were summarized by standard descriptive statistics. The associations between NGAL expression and clinicopathologic variabels as well as associations with pCR in various subgroups were assessed by cross-tabulation, x^2^-test and Fisher's exact test. Cox proportional hazard models were used to perform uni- and multivariate analysis and to determine the prognostic significance of the reviewed variables, including NGAL expression, for being predictive markers for pCR, DFS and OS. DFS and OS survival probabilities were estimated using the Kaplan-Meier product limit method. Log rank tests were used to calculate the survival functions. No correction for multiple testing was performed. P-values≤0.05 were considered as statistically significant. For statistical analysis of data, the Software packages SPSS 14.0 and SAS 9.2 were used. All tests were two-sided.

## Results

### NGAL expression in breast carcinoma cells

Pretreatment breast cancer biopsies from 855 participants of the GeparTrio trial were evaluated for NGAL expression. Due to loss of tumor tissue during tissue microarray construction, NGAL labelling was interpretable in 651 breast cancer samples. In 42.2% (n = 275) of the breast cancer patients NGAL detection was positive. Positive immunoreactivity was predominantly detected in the cytoplasm, but a subset of carcinomas showed secretion of NGAL in the duct lumens. NGAL staining intensity varied from negative to strong staining (Figure 1). 375 (57.7%) tumors presented negative staining, 169 (26.1%) showed a weak intensity. Medium intensity was expressed by 61 (9.4%) patients and 44 (6.8%) patients had strong staining intensity. The distribution pattern of NGAL labelling was comparable to our previous findings and varied from a weak staining of all tumor cells to a strong focal labelling [5]. 12 patients (1.8%) showed strong NGAL expression in all tumor cells. Remarkably, only 4% (n = 11) of the NGAL positive tumors were lobular carcinomas, whereas 96% (n = 264) showed ductal or other histological subtypes.

### Association between NGAL expression and clinicopathologic parameters

NGAL expression in breast carcinoma cells was significantly associated with histological tumor type, hormone receptor (HR) status, estrogen receptor (ER) and progesterone receptor (PR) status.

Positive NGAL labelling was significantly associated with the ductal or other histological subtypes (p = 0.009). Positive NGAL expression and strong staining intensity (3) were correlated with steroid receptor status. Negative receptor status was significantly more prevalent in NGAL positive tumors than in NGAL negative tumors (Table 1). The proportion of tumors with negative hormone receptor status was also higher in tumors with an NGAL intensity score of 3 compared to tumors with intensity scores of 2, 1 or 0 (Table 1). No significant correlations were found between NGAL expression or intensity and HER2 status, nodal status, histological grade, tumor size and age (Table 1).

**Table 1:** Association between NGAL-expression and clinicopathologic variables.

Variable	NGAL expression (%)		p-value	NGAL staining intensity (%)				p-value
	positive	negative		0	1	2	3	
**Hormone receptor status (n = 639)**								
*positive (n = 487)*	71.9	79.3	**0.038**	79.2	76.1	73.3	53.5	**0.002**
*negative (n = 152)*	28.1	20.7		20.8	23.9	26.7	46.5	
**Estrogen receptor status (n = 640)**								
*positive (n = 487)*	71.6	79.3	**0.031**	79.2	75.6	73.3	53.5	**0.002**
*negative (n = 153)*	28.4	20.7		20.8	24.4	26.7	46.5	
**Progesterone receptor status (n = 619)**								
*positive (n = 343)*	50.6	58.8	**0.049**	58.7	57.4	46.6	30.2	**0.002**
*negative (n = 276)*	49.4	41.2		41.3	42.6	53.4	69.8	
**HER2 status (n = 640)**								
*positive (n = 135)*	21.1	21.1	1.0	21.1	21.0	19.0	22.7	0.973
*negative (n = 505)*	78.9	78.9		78.9	79.0	81.0	77.3	
**Histological grade (n = 650)**								
*1/2 (n = 491)*	74.1	76.6	0.462	76.5	79.3	65.6	65.9	0.073
*3 (n = 159)*	25.9	23.4		23.5	20.7	34.4	34.1	
**Tumor type (n = 651)**								
*ductal and other (n = 605)*	96.0	90.7	**0.009**	90.7	96.4	93.4	97.7	0.054
*lobular (n = 46)*	4.0	9.3		9.3	3.6	6.6	2.3	
**Lymph node status (n = 630)**								
*positive (n = 346)*	56.3	53.9	0.572	53.9	54.3	60.7	59.1	0.729
*negative (n = 284)*	43.7	46.1		46.1	45.7	39.3	40.9	
**Tumor size [mm] (n = 637)**								
*<40 (n = 261)*	39.3	42.2	0.515	42.3	39.8	37.7	40.9	0.889
*≥40 (n = 376)*	60.7	57.8		57.7	60.2	62.3	59.1	
**Age [years] (n = 642)**								
*<50 (n = 297)*	44.0	48.0	0.337	47.8	41.9	49.1	45.5	0.604
*≥50 (n = 345)*	56.0	52.0		52.2	58.1	50.8	54.5	

Abbreviations: NGAL, Neutrophil gelatinase-associated lipocalin; HER2, human epidermal growth factor receptor 2.

### Association between NGAL expression and pCR

Overall pCR rate was 21% in the entire cohort. pCR rate in NGAL negative patients was 19.8% and pCR rate in NGAL positive patients 22.7%. If we use the staining intensity, the group with strong NGAL staining had a pCR rate of 31.8% compared to 19.7% in the group with moderate staining and 21.6% in patients with weak NGAL staining intensity. NGAL expression was significantly associated with higher pCR rates in patients with positive hormone receptor status (p = 0.033). In patients with hormone receptor positive tumors the pCR rate in NGAL positive tumors was 18.4% vs. 11.3% in NGAL negative tumours. NGAL was shown to be a marker for lower pCR rates in hormone receptor negative patients (30.7% vs. 51.4%, p = 0.013). Accordant results have been shown for patients with positive and negative estrogen receptor status (Table 2). In the categories PR status, HER2 status, histological grade, lymph node status, tumor type and size as well as age no association between NGAL expression and pCR rate was detected.

**Table 2:** Association between NGAL expression and pCR.

Variable		NGAL expression (%)		p-value	NGAL staining intensity (%)				p-value
		positive	negative		0	1	2	3	
**Entire cohort (n = 642)**	pCR	22.7	19.8	0.379	19.8	21.6	19.7	31.8	0.323
	no pCR	77.3	80.2		80.2	78.4	80.3	68.2	
**Hormone receptor status (n = 639)**									
*positive (n = 487)*	pCR	18.4	11.3	**0.033**	11.4	14.8	20.5	34.8	**0.010**
	no pCR	81.6	88.7		88.6	85.2	79.5	65.2	
*negative (n = 152)*	pCR	30.7	51.4	**0.013**	51.4	41.0	12.5	25.0	**0.013**
	no pCR	69.3	48.6		48.6	59.0	87.5	75.0	
**Estrogen receptor status (n = 640)**									
*positive (n = 487)*	pCR	18.4	11.3	**0.033**	11.4	14.8	20.5	34.8	**0.010**
	no pCR	81.6	88.7		88.6	85.2	79.5	65.2	
*negative (n = 153)*	pCR	30.3	51.4	**0.012**	51.4	40.0	12.5	25.0	**0.013**
	no pCR	69.7	48.9		48.6	60.0	87.5	75.0	
**Progesterone receptor status (n = 619)**									
*positive (n = 343)*	pCR	10.8	10.8	1.0	10.8	9.0	11.1	23.1	0.507
	no pCR	89.2	89.2		89.2	91.0	88.9	76.9	
*negative (n = 276)*	pCR	32.3	32.2	1.0	32.2	36.4	22.6	33.3	0.603
	no pCR	67.7	67.8		67.8	63.6	77.4	66.7	
**HER2 status (n = 640)**									
*positive (n = 135)*	pCR	36.8	28.9	0.355	28.9	40.0	27.3	40.0	0.631
	no pCR	63.2	71.1		71.1	60.0	72.7	60.0	
*negative (n = 505)*	pCR	18.9	17.1	0.637	17.1	16.8	17.0	29.4	0.345
	no pCR	81.1	82.9		82.9	83.2	83.0	70.6	
**Histological grade (n = 650)**									
*1/2 (n = 491)*	pCR	19.9	16.3	0.335	16.4	16.7	17.5	37.9	**0.036**
	no pCR	80.1	83.7		83.6	83.3	82.5	62.1	
*3 (n = 159)*	pCR	31.0	31.0	1.0	31.0	40.0	23.8	20.0	0.443
	no pCR	69.0	69.0		69.0	60.0	76.2	80.0	
**Tumor type (n = 651)**									
*ductal and other (n = 605)*	pCR	23.7	21.3	0.490	21.3	22.4	21.1	32.6	0.418
	no pCR	76.3	78.7		78.7	77.6	78.9	67.4	
*lobular (n = 46)*	pCR	0.0	5.7	1.0	5.7	0.0	0.0	0.0	0.883
	no pCR	100.0	94.3		94.3	100.0	100.0	100.0	
**Lymph node status (n = 630)**									
*positive (n = 346)*	pCR	19.7	20.1	1.0	20.1	20.2	18.9	19.2	0.998
	no pCR	80.3	79.9		79.9	79.8	81.1	80.8	
*negative (n = 284)*	pCR	27.1	19.9	0.156	19.9	24.0	20.8	50.0	**0.038**
	no pCR	72.9	80.1		80.1	76.0	79.2	50.0	
**Tumor size [mm] (n = 637)**									
*<40 (n = 261)*	pCR	25.5	21.1	0.452	21.1	22.7	13.6	50.0	**0.031**
	no pCR	74.5	78.9		78.9	77.3	86.4	50.0	
*≥40 (n = 376)*	pCR	20.0	18.0	0.691	18.1	20.0	21.1	19.2	0.963
	no pCR	80.0	82.0		81.9	80.0	78.9	80.0	
**Age [years] (n = 642)**									
*<50 (n = 297)*	pCR	31.7	24.3	0.185	24.4	31.4	26.7	40.0	0.398
	no pCR	68.3	75.7		75.6	68.6	73.3	60.0	
*≥50 (n = 345)*	pCR	15.7	15.6	1.0	15.6	14.4	12.9	25.0	0.600
	no pCR	84.3	84.4		84.4	85.6	87.1	75.0	

Abbreviations: NGAL, Neutrophil gelatinase-associated lipocalin; pCR, pathologic complete response; HER2, human epidermal growth factor receptor 2.

NGAL staining intensity was shown to be a marker for higher pCR rates in several subgroups of known low risk, such as estrogen and progesterone positive and lymph node negative patients, patients with histological grade 1 or 2 tumors and a tumor size <40 mm (Table 2). In the HR and ER positive subgroups higher pCR rates were detected in tumors with a NGAL intensity score of 3 compared to tumors with lower intensity scores of 2, 1 or 0 34.8% vs. 20.5% vs. 14.8% vs. 11.4%, p = 0.01). Furthermore, the subgroup of lymph node negative patients presented higher pCR rates in tumors with strongest NGAL intensity (50.0% vs. 20.8% vs. 24.0% vs. 19.9%, p = 0.038). Within the group of patients with favourable grading the proportion of tumors with higher pCR rates was higher in tumors showing strongest NGAL staining than in those with less staining intensity (37.9% vs. 17.5% vs. 16.7% vs. 16.4%, p = 0.036). Finally, regarding the group of tumors with a size <40 mm, higher pCR rates were found in the intensity 3 tumors compared to tumors with a staining intensity of 2, 1 or 0 (50.0% vs. 13.6% vs. 22.7% vs. 21.1%, p = 0.031). In addition, strong NGAL staining intensity was associated with higher pCR rates in further groups of low-risk such as PR positive and HER2 negative tumors, tumors of ductal type and in patients younger than 50 years of age. However, these results did not reach significance.

Multivariate cox regression analysis revealed age, HR and HER2 status as independent predictors of pCR. NGAL failed to be an independent predictor of pCR in the entire study cohort (data not shown).

### Association between NGAL expression and disease-free survival

In univariate survival analysis, positive NGAL expression and strong NGAL staining intensity in breast carcinoma cells were highly significantly associated with decreased DFS in the entire cohort (NGAL expression: HR = 1.82, p<0.001; NGAL intensity: HR = 2.16, p<0.001). Mean DFS was 77.1 months in the entire group. NGAL negative patients showed a mean DFS of 81.5 months, whereas the NGAL positive group had a mean DFS of 67.0 months.

Stratification into different subgroups revealed a significant association between NGAL expression and DFS in multiple groups. Decreased DFS in NGAL expressing tumors was found in patients who fail to respond to NACT with pCR (HR = 2.12, p<0.001), in patients with positive HR and ER status (HR = 1.91, p = 0.002) and both positive and negative PR expressing patients (PR positive: HR = 2.12, p = 0.003; PR negative: HR = 1.67, p = 0.038). Similar results were achieved for HER2 negative tumors (HR = 1.92, p = 0.001), lymph node positive tumors (HR = 2.07, p<0.001), grade 1 and 2 tumors (HR = 2.04, p<0.001), the ductal subtype (HR = 1.8, p = 0.001), a tumor size <40 mm (HR = 1.85, p = 0.043) as well as ≥40 mm (HR = 1.83, p = 0.005) and patients older than 50 years (HR = 2.23, p<0.001) (Table 3).

**Table 3:** Univariate Cox Survival Analysis for NGAL expression.

Variable	NGAL expression	Disease-free survival				Overall survival			
		E/N	Hazard ratio	95% confidence interval	p-value	E/N	Hazard ratio	95% confidence interval	p-value
**Entire cohort**	positive	73/273	1.00	1.294–2.556	**<0.001**	37/273	1.00	0.881–2.161	0.159
	negative	61/369	1.82			40/369	1.38		
**pCR**									
*yes*	positive	7/62	1.00	0.316–2.103	0.673	4/62	1.00	0.234–2.936	0.771
	negative	11/73	0.82			6/73	0.83		
*no*	positive	66/211	1.00	1.464–3.059	**<0.001**	33/211	1.00	0.933–2.440	0.093
	negative	50/296	2.12			34/296	1.51		
**Hormone receptor status**									
*positive*	positive	47/190	1.00	1.262–2.892	**0.002**	24/190	1.00	0.846–2.526	0.174
	negative	43/291	1.91			28/291	1.46		
*negative*	positive	23/78	1.00	0.732–2.568	0.324	12/75	1.00	0.456–2.261	0.971
	negative	17/74	1.37			12/74	1.02		
**Estrogen receptor status**									
*positive*	positive	47/190	1.00	1.262–2.892	**0.002**	24/190	1.00	0.846–2.526	0.174
	negative	43/291	1.91			28/291	1.46		
*negative*	positive	24/76	1.00	0.760–2.637	0.273	12/76	1.00	0.448–2.221	0.995
	negative	17/74	1.42			12/74	0.99		
**Progesterone receptor status**									
*positive*	positive	33/130	1.00	1.282–3.501	**0.003**	15/130	1.00	0.734–2.858	0.285
	negative	29/213	2.12			19/213	1.45		
*negative*	positive	38/127	1.00	1.028–2.705	**0.038**	21/127	1.00	0.682–2.323	0.462
	negative	29/146	1.67			21/146	1.26		
**HER2 status**									
*positive*	positive	20/57	1.00	0.850–3.105	0.142	8/57	1.00	0.400–2.475	0.990
	negative	17/76	1.63			12/76	0.99		
*negative*	positive	53/212	1.00	1.285–2.879	**0.001**	29/212	1.00	0.964–2.757	0.068
	negative	43/285	1.92			27/287	1.63		
**Histological grade**									
*1 and 2*	positive	51/201	1.00	1.345–3.085	**<0.001**	27/201	1.00	0.907–2.649	0.109
	negative	40/282	2.04			27/282	1.55		
*3*	positive	22/71	1.00	0.711–2.388	0.392	10/71	1.00	0.440–2.291	0.993
	negative	21/77	1.30			13/87	1.00		
**Tumor type**									
*ductal and other*	positive	71/262	1.00	1.268–2.548	**0.001**	35/262	1.00	0.869–2.210	0.171
	negative	57/334	1.80			36/334	1.39		
*lobular*	positive	2/11	1.00	0.272–8.241	0.643	2/11	1.00	0.328–11.798	0.458
	negative	4/35	1.50			4/35	1.97		
**Lymph node status**									
*positive*	positive	53/152	1.00	1.355–3.163	**<0.001**	28/152	1.00	0.840–2.446	0.186
	negative	36/194	2.07			26/194	1.43		
*negative*	positive	20/118	1.00	0.771–2.562	0.266	9/118	1.00	0.490–2.769	0.729
	negative	23/166	1.41			12/166	1.17		
**Tumor size [mm]**									
*<40*	positive	22/106	1.00	1.019–3.374	**0.043**	15/106	1.00	1.090–5.170	**0.029**
	negative	21/154	1.85			12/152	2.37		
*≥40*	positive	51/165	1.00	1.199–2.782	**0.005**	22/165	1.00	0.563–1.741	0.971
	negative	38/211	1.83			27/211	0.99		
**Age [years]**									
*<50*	positive	29/120	1.00	0.865–2.382	0.162	16/120	1.00	0.650–2.464	0.488
	negative	31/177	1.44			19/177	1.27		
*≥50*	positive	44/153	1.00	1.399–3.552	**<0.001**	21/153	1.00	0.862–3.000	0.136
	negative	30/192	2.23			21/192	1.61		

Abbreviations: NGAL, Neutrophil gelatinase-associated lipocalin; pCR, pathologic complete response; HER2, human epidermal growth factor receptor 2; E, number of events; N, total sample size.

**Figure 2:**
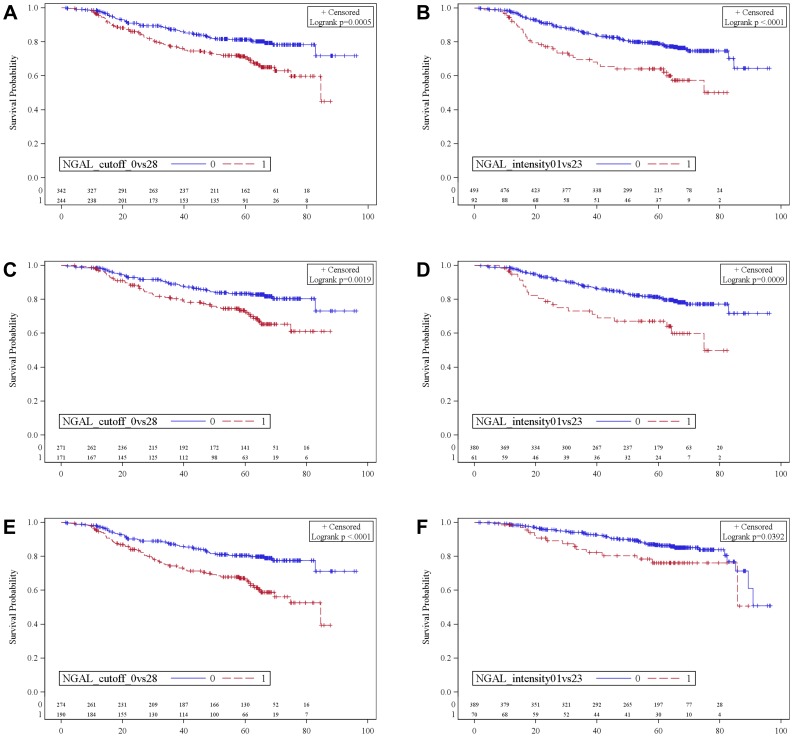
Long-term outcome of patients depending on NGAL expression and intensity in months. (**A**) DFS in all patients by NGAL expression neg. vs. pos. (**B**) DFS in all patients by NGAL intensity 0–1 vs. 2–3. (**C**) DFS in HR positive patients by NGAL expression neg. vs. pos. (**D**) DFS in HR positive patients by NGAL intensity 0–1 vs. 2–3. (**E**) DFS in patients without pCR by NGAL expression neg. vs. pos. (**F**) OS in patients without pCR by NGAL intensity 0–1 vs. 2–3. NGAL: neutrophil gelatinase-associated lipocalin. DFS: disease-free survival. OS: overall survival. HR: hormone.

In these subgroups consistent results were observed for strong NGAL staining intensity. Tumors that feature a NGAL intensity of 2 or 3 were significantly associated with shorter DFS than tumors with a NGAL intensity 0 or 1 (Table 4). The only groups that have to be added to the list above are patients with an unfavourable grading (G3; HR = 2.12, p = 0.003) and negative lymph node status (HR = 2.34, p = 0.02), they also show decreased DFS when having a strong NGAL staining intensity (Figure 2 A–F).

**Table 4:** Univariate Cox Survival Analysis for NGAL staining intensity.

Variable	NGAL staining intensity	Disease-free survival				Overall survival			
		E/N	Hazard ratio	95% confidence interval	p-value	E/N	Hazard ratio	95% confidence interval	p-value
**Entire cohort**	0 and 1	100/535	1.00	1.462–3.192	**<0.001**	60/535	1.00	0.984–2.898	0.057
	2 and 3	34/105	2.16			17/105	1.69		
**pCR**									
*yes*	0 and 1	15/109	1.00	0.291–3.476	0.992	8/109	1.00	0.255–5.664	0.816
	2 and 3	3/26	1.01			2/26	1.20		
*no*	0 and 1	85/426	1.00	1.669–3.815	**<0.001**	52/426	1.00	1.021–3.234	**0.042**
	2 and 3	31/79	2.52			15/79	1.82		
**Hormone receptor status**									
*positive*	0 and 1	69/412	1.00	1.379–3.672	**0.001**	41/412	1.00	0.949–3.615	0.071
	2 and 3	21/67	2.25			11/67	1.85		
*negative*	0 and 1	27/113	1.00	0.995–3.778	0.0516	18/113	1.00	0.494–3.145	0.6408
	2 and 3	13/36	1.934			6/36	1.25		
**Estrogen receptor status**									
*positive*	0 and 1	69/412	1.00	1.379–3.672	**0.0012**	41/412	1.00	0.949–3.615	0.0706
	2 and 3	21/67	2.25			11/67	1.85		
*negative*	0 and 1	28/114	1.00	0.978–3.681	0.0583	18/114	1.00	0.500–3.181	0.6238
	2 and 3	13/36	1.90			6/36	1.26		
**Progesterone receptor status**									
*positive*	0 and 1	48/301	1.00	1.461–4.824	**0.0014**	26/301	1.00	1.249–6.154	**0.0122**
	2 and 3	14/40	2.66			8/40	2.77		
*negative*	0 and 1	47/212	1.00	1.087–3.111	**0.0231**	33/212	1.00	0.531–2.336	0.7764
	2 and 3	20/61	1.84			9/61	1.11		
**HER2 status**									
*positive*	0 and 1	29/111	1.00	0.740–3.566	0.220	17/111	1.00	0.269–3.208	0.906
	2 and 3	8/21	1.62			3/21	0.93		
*negative*	0 and 1	70/417	1.00	1.541–3.803	**<0.001**	42/417	1.00	1.141–3.847	**0.017**
	2 and 3	26/81	2.42			14/81	2.10		
**Histological grade**									
*1 and 2*	0 and 1	70/413	1.000	1.292–3.433	**0.003**	45/413	1.00	0.644–2.703	0.449
	2 and 3	21/69	2.12			9/69	1.32		
*3*	0 and 1	30/122	1.00	1.052–3.917	**0.035**	15/122	1.00	0.932–5.208	0.072
	2 and 3	13/36	2.03			8/36	2.20		
**Tumor type**									
*ductal and other*	0 and 1	95/494	1.00	1.461–3.234	**<0.001**	56/494	1.00	0.887–2.781	0.121
	2 and 3	33/100	2.17			15/100	1.57		
*lobular*	0 and 1	5/41	1.00	0.188–14.063	0.659	4/41	1.00	0.834–30.048	0.078
	2 and 3	1/5	1.62			2/5	5.01		
**Lymph node status**									
*positive*	0 and 1	65/283	1.00	1.220–3.115	**0.005**	42/283	1.00	0.752–2.715	0.276
	2 and 3	24/63	1.95			12/63	1.43		
*negative*	0 and 1	33/241	1.00	1.145–4.772	**0.020**	16/241	1.00	0.727–5.490	0.1796
	2 and 3	10/42	2.34			5/42	1.99		
**Tumor size [mm]**									
*<40*	0 and 1	32/218	1.00	1.413–5.613	**0.003**	21/218	1.00	0.881–5.482	0.091
	2 and 3	11/40	2.82			6/40	2.20		
*≥40*	0 and 1	66/310	1.00	1.168–3.024	**0.009**	38/310	1.00	0.695–2.680	0.366
	2 and 3	23/64	1.88			11/64	1.37		
**Age [years]**									
*<50*	0 and 1	46/246	1.00	0.926–3.067	0.087	26/246	1.00	0.839–3.836	0.131
	2 and 3	14/50	1.69			9/50	1.79		
*≥50*	0 and 1	54/289	1.00	1.590–4.474	**<0.001**	34/289	1.00	0.763–3.604	0.202
	2 and 3	20/55	2.67			8/55	1.66		

Abbreviations: NGAL: Neutrophil gelatinase-associated lipocalin. pCR: pathologic complete response. HER: human epidermal growth factor receptor; E, number of events; N, total sample size.

### Association between NGAL expression and overall survival

A statistically significant association between decreased OS and positive NGAL expression was shown in patients with a tumor size <40 mm (HR = 2.37, p = 0.029) in univariate cox survival analysis (Table 3). Patients with strong NGAL staining intensity (2 or 3) had a significantly decreased OS compared to tumors with NGAL staining 0 or 1 if they fail to reach pCR (HR = 1.82, p = 0.042; Figure 2 E). The same applied on patients in the PR positive (HR = 2.77, p = 0.012) and in the HER2 negative subgroup (HR = 2.1, p = 0.017; Table 4).

### Multivariate survival analysis

Known independent markers for decreased DFS and OS could be confirmed in multivariate cox proportional hazard analysis (Table 5 and 6). DFS was independently marked by pCR (HR = 0.4, p<0.001), hormone receptor status (HR = 0.64, p = 0.038) and nodal status (HR = 1.69, p = 0.007), HER2 status (HR = 0.66, p = 0.038) and histological grade (HR = 1.62, p = 0.021) (Table 5). The following variables were identified as independent prognostic markers for OS: pCR (HR = 0.41, p = 0.015), hormone receptor status (HR = 0.56, p = 0.04) and nodal status (HR = 2.21, p = 0.003). Positive NGAL expression (HR = 1.76; p = 0.002) and strong NGAL intensity (HR = 2.05; p = 0.004) were independently prognostic for decreased DFS in multivariate analysis. For OS, NGAL expression and intensity failed to be identified as independent prognostic factors (Table 6).

**Table 5:** Multivariate Cox Survival Analysis - Disease-free survival.

Variable	Hazard ratio	95% confidence interval	p-value
**NGAL expression**			
*positive*	1.76	1.230–2.522	**0.002**
*negative*			
**NGAL staining intensity**			
*2 and 3*	2.05	1.254–3.362	**0.004**
*0 and 1*			
**pCR**			
*yes*	0.40	0.234–0.682	**<0.001**
*no*			
**Hormone receptor status**			
*positive*	0.64	0.418–0.975	**0.038**
*negative*			
**HER2 status**			
*negative*	0.66	0.442–0.977	**0.038**
*positive*			
**Histological grade**			
*3*	1.62	1.075–2.426	**0.021**
*1 and 2*			
**Tumor type**			
*ductal/other*	0.68	0.272–1.708	0.413
*lobular*			
**Lymph node status**			
*positive*	1.69	1.152–2.468	**0.007**
*negative*			
**Tumor size [mm]**			
*≥40*	1.14	0.773–1.687	0.505
*<40*			
**Age [years]**			
*≥50*	0.94	0.653–1.345	0.726
*<50*			

**Table 6:** Multivariate Cox Survival Analysis - Overall survival.

Variable	Hazard ratio	95% confidence interval	p-value
**NGAL expression**			
*positive*	1.35	0.842–2.159	0.213
*negative*			
**NGAL staining intensity**			
*2 and 3*	1.40	0.754–2.594	0.288
*0 and 1*			
**pCR**			
*yes*	0.41	0.198–0.843	**0.015**
*no*			
**Hormone receptor status**			
*positive*	0.56	0.323–0.973	**0.040**
*negative*			
**HER2 status**			
*negative*	0.79	0.463–1.337	0.376
*positive*			
**Histological grade**			
*3*	1.58	0.910–2.725	0.105
*1 and 2*			
**Tumor type**			
*lobular*	1.44	0.554–3.736	0.4549
*ductal/other*			
**Lymph node status**			
*positive*	2.21	1.310–3.737	**0.003**
*negative*			
**Tumor size [mm]**			
*<40*	0.99	0.604–1.654	0.998
*≥40*			
**Age [years]**			
*≥50*	0.91	0.569–1.463	0.703
*<50*			

## Discussion

In this study we could evaluate NGAL as a potentially predictive marker for response to NACT in low-risk groups of primary human breast cancer and validate NGAL as a predictor of poor prognosis in this entity. NGAL expression was positive in 42.2% of all cases. This number lies within the range of previous reports [5], [17], [36].

Our findings show an association between NGAL expression and negative hormone receptor status (ER and PR). This confirms the results of our previous work [5] and is also consistent with the statements of other studies, that examined gene expression profiling of breast carcinomas [37], [38]. Negative hormone receptor status is known to be a parameter for more aggressive tumors, which are characterized by showing better response to NACT, more often achieving pathological complete response [39], [40]. Like negative hormone receptor status there exist several high-risk biological markers predicting pCR [39]. In contrast, there are no biological markers predicting pCR in low-risk groups. The question if patients with low-risk tumor characteristics would benefit from an additional chemotherapy is often raised in daily clinical routine. So far there are no instruments easy to access that would help us in decision-making. One commercially avaible gene expression test was introduced to evaluate patients' individual risk of relapse and response to chemotherapy, guiding the way for introducing patients with a low-risk tumor profile to chemotherapy or not. Drawbacks of the use of this testing module for clinical routine are the effort of time and costs [41].

The present results reveal NGAL staining intensity as a marker for higher pCR rates in subgroups of low risk. This applies on hormone receptor positive and node negative patients, patients with favourable histological grade and a tumor size <40 mm. This is a very interesting and useful finding, especially in the context mentioned above. Nevertheless, it also has to be recognized that NGAL did not show to be an independent predictor for pCR in multivariate analysis.

In survival analysis, decreased DFS in patients whose tumors showed positive NGAL expression was found in the entire cohort, but also in different subgroups. Those were patients without pCR, with positive HR and ER status, positive and negative PR expressing tumors, HER2 negative and lymph node positive tumors, grade 1 and 2 tumors, the ductal subtype, a tumor size <40 mm as well as ≥40 mm and patients older than 50 years. Decreased OS in NGAL expressing patients was only noted in patients with a tumor smaller than 40 mm, respectively in patients who failed to reach pCR or with HER2 negative or PR positive tumors regarding NGAL intensity.

Inspite of the large sample size of tumors in this study, NGAL failed to be an independent marker for OS, as it has also done previously [5]. But NGAL expression and intensity were shown to be independent predictors for DFS, which confirms our former findings [5]. Thus, not only does NGAL expression present itself as a predictor for response to chemotherapy in subgroups of low risk, it also appears to be a marker for recurrence of disease. Therefore, it is conceivable that NGAL might be a future marker for individual therapeutic decisions to enable a tailored therapy for each breast cancer patient. NGAL expression can be easily determined by immunohistochemistry in daily routine. No additional tissue sampling is necessary.

For a methodical point our results are based on reliable data due to the homogeneous collective underlying the German GeparTrio study. In this large study cohort each patient met the same inclusion criteria and received identical chemotherapy regime. However, it has to be borne in mind that validation of predictive markers strongly depends on the drugs used in the specific therapeutic setting [39].

The NGAL molecule plays an important role in cell biology and interfears with different molecular pathways. Its functions can be divided into pro-tumoral and anti-tumoral effects. For pro-tumoral effect NGAL participates in the intracellular capture of iron [42]. Furthermore, NGAL assists tumor growth and angiogenesis by forming complexes with MMP-9, thereby protecting MMP-9 from degradation [43]. Additionally, NGAL plays a role in the mechanisms of estrogen-induced growth. [42]. NGAL has an anti-metastatic role by inhibiting HIF-1α factor, FA-Kinase phosphorylation and also by retaining synthesis of vascular endothelial growth factor (VEGF) [42].

In summary, NGAL was found to be a predictive marker for pCR after NACT in low-risk subgroups. Furthermore, NGAL could be validated as an independent prognostic factor for decreased DFS in primary human breast cancer. To realise an individualized targeted therapy for breast cancer patients further knowledge and reliability concerning predictive markers for chemotherapy are necessary. Nevertheless, NGAL appears to be a very promising part on the way to achieve this goal.
